# Pro-inflammatory intestinal Th17-cells are tissue-resident, accumulate in the epithelia in Crohn’s disease, and predict unresponsiveness to vedolizumab

**DOI:** 10.1093/ecco-jcc/jjag055

**Published:** 2026-06-09

**Authors:** Moira Paroni, Carmen Faggiano, Camilla Righetti, Irene Dusetti, Valeria Ranzani, Giorgia Moschetti, Daniele Noviello, Chiara Vasco, Maurizio Vecchi, Paolo Landini, Federica Facciotti, Sergio Abrignani, Flavio Caprioli, Jens Geginat

**Affiliations:** Department of Biosciences, Dipartimento di Eccellenza 2023-2027, Università degli Studi di Milano, Milan, Italy; National Institute of Molecular Genetics (INGM) “Romeo ed Enrica Invernizzi”, Milan, Italy; National Institute of Molecular Genetics (INGM) “Romeo ed Enrica Invernizzi”, Milan, Italy; Department of Biosciences, Dipartimento di Eccellenza 2023-2027, Università degli Studi di Milano, Milan, Italy; National Institute of Molecular Genetics (INGM) “Romeo ed Enrica Invernizzi”, Milan, Italy; National Institute of Molecular Genetics (INGM) “Romeo ed Enrica Invernizzi”, Milan, Italy; Gastroenterology and Endoscopy Unit, Fondazione IRCCS Ca’ Granda Ospedale Maggiore Policlinico, Milan, Italy; Department of Pathophysiology and Transplantation, Università degli Studi di Milano, Milan, Italy; National Institute of Molecular Genetics (INGM) “Romeo ed Enrica Invernizzi”, Milan, Italy; Gastroenterology and Endoscopy Unit, Fondazione IRCCS Ca’ Granda Ospedale Maggiore Policlinico, Milan, Italy; Department of Pathophysiology and Transplantation, Università degli Studi di Milano, Milan, Italy; Department of Biosciences, Dipartimento di Eccellenza 2023-2027, Università degli Studi di Milano, Milan, Italy; Department Biotechnology and Biosciences, Università Milano-Bicocca, Milan, Italy; National Institute of Molecular Genetics (INGM) “Romeo ed Enrica Invernizzi”, Milan, Italy; DISCCO, Department of Clinical Science and Community Health, Dipartimento di Eccellenza 2023-27, Università degli studi di Milano, Milan, Italy; Gastroenterology and Endoscopy Unit, Fondazione IRCCS Ca’ Granda Ospedale Maggiore Policlinico, Milan, Italy; Department of Pathophysiology and Transplantation, Università degli Studi di Milano, Milan, Italy; National Institute of Molecular Genetics (INGM) “Romeo ed Enrica Invernizzi”, Milan, Italy; DISCCO, Department of Clinical Science and Community Health, Dipartimento di Eccellenza 2023-27, Università degli studi di Milano, Milan, Italy

**Keywords:** Th17-cells, intraepithelial lymphocytes, vedolizumab

## Abstract

We recently identified a population of pro-inflammatory intestinal CCR5^+^Th17-cells (proinflammatory Th17, pTh17) associated with intestinal inflammation in Crohn’s Disease (CD) and activated by CD-associated adherent-invasive *Escherichia coli*. Here, we report that intestinal pTh17-cells in CD expressed genes characteristic of tissue-resident memory T-cells (T_RM_). They expressed high levels of CD69 and CD103 on the cell surface and accumulated in the intraepithelial lymphocyte (IEL) compartment in CD. Consistently, single T-cell RNA sequencing in CD unveiled that intraepithelial Th17-cells displayed a gene signature of activated pTh17-cells.In peripheral blood, pTh17-cells with gut-homing potential were hardly detectable. Conversely, conventional CCR5^-^Th17-cells (“cTh17”) were present and expressed increased levels of α4β7-integrin in the blood of IBD patients. Consistently, intestinal cTh17-cells were reduced after treatment with vedolizumab, which blocks α4β7-integrin-mediated gut-homing of lymphocytes. Importantly, unresponsiveness to vedolizumab in CD was associated with increased frequencies of preexisting intestinal pTh17-cells with high sensitivity and specificity. We conclude that intestinal pTh17-cells are tissue-resident cells, which could be generated from circulating cTh17 precursor cells in the gut. Moreover, our findings are consistent with a key pathogenic role of intraepithelial Th17-cells in CD and suggest that monitoring intestinal pTh17-cells may predict unresponsiveness to vedolizumab.

## Graphical abstract

**Graphical Abstract:**
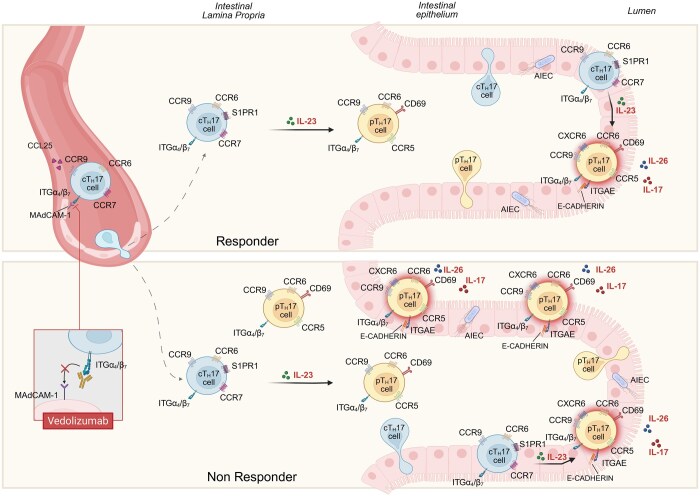

## 1. Introduction

Crohn’s disease (CD) is characterized by chronic intestinal inflammation, a dysbiotic gut microbiota and the expansion of potentially pathogenic IFN-γ and IL-17 co-producing CD4^+^T-cells.^1^ We recently reported that a highly proinflammatory population of CCR5^+^Th17 effector cells (named “pTh17”) was associated with intestinal inflammation in CD and ­produced proinflammatory cytokines in response to adherent-invasive *Escherichia coli* (AIEC), suggesting that they contribute to drive intestinal inflammation in CD.^2^ pTh17-cells could be induced by IL-23 from conventional, CCR5^-^Th17-cells (cTh17-cells) in vitro, and were reduced after treatment with the therapeutic anti-IL-23p19 monoclonal antibody risankizumab^3^ in the intestinal lamina propria (LP) of CD patients. Notably, pTh17-cells lack CXCR3 expression and produce high levels of type 17 cytokines. They are thus distinct from the well-established CCR6^+^CXCR3^+^Th1/17-cells that produce predominantly IFN-γ and low levels of type 17 cytokines.^4^^,^^5^

Tissue-resident-memory T cells (T_RM_),^6^ a peculiar population of T-cells that express CD103 and/or CD69, were reported to be enriched in the intestinal mucosa of IBD patients and to produce pro-inflammatory cytokines, including IL-17.^7^^,^^8^ T_RM_ cells permanently reside in peripheral tissues, including the gut. They may provide immediate protection,^7^ but they could also contribute to intestinal inflammation in IBDs.^8^ Since T_RM_ do not recirculate, we hypothesized that they may represent an obstacle for IBD therapies that target T-cell migration to the gut like the anti-α4β7 antibody vedolizumab. Here, we report that intestinal pTh17-cells are T_RM_ that home to the epithelium and are indeed associated with unresponsiveness to vedolizumab.

## 2. Methods

### Patients

All IBD patients included in this study were classified according to clinical and endoscopic disease activity, using Harvey–Bradshaw index (HBI) and the Simple Endoscopic Score (SES-CD) for CD patients, respectively, or Mayo score for UC patients (Table 1). For some experiments, biopsies were taken before and after treatment with vedolizumab. Vedolizumab was given intravenously at a fixed dose of 300 mg at weeks 0, 2, 6, 10, 14, and then every 8 weeks. The clinical characteristics of untreated and vedolizumab-treated patients were reported previously.^2^^,^^9^ In all patients, a baseline biopsy was performed in the area of the most severe endoscopic inflammation. Posttreatment biopsies were taken specifically targeting the same area. The bottom of ulcers or areas suspected of being dysplastic were specifically avoided. Endoscopic response to vedolizumab was defined as a decline in SES-CD score of at least 50%. The study was registered with Eudract ref. no. 2015-003270-32 and the institutional review board approved the study with permission no. 566_2015 quinqies. The study was performed in accordance with the Declaration of Helsinki protocols.

**Table 1.** Baseline clinical characteristics of the vedolizumab-treated IBD patients.

Clinical parameter	Crohn’s disease (*n* = 18)	Ulcerative colitis (*n* = 20)	*P*
**Male/female, *n***	14/4	12/8	.6851
**Age at diagnosis, mean ± SD (years)**	25 ± 9	36 ± 14	.01732
**Age at enrolment, mean ± SD (years)**	42 ± 13	45 ± 15	.69654
**Disease duration, ± SD (years)**	16 ± 10	9 ± 7	.00854
**Smoking status, yes/no**	4/14	1/19	.276774
**Disease location^a^ (ulcerative colitis), *n* ^a^**			**–**
** E1 (proctitis)**	/	0	
** E2 (left sided)**	/	10	
** E3 (extensive)**	/	10	
**Disease locatio**n**^a^ (Crohn’s disease), *n* ^a^**			**–**
** L1 (ileal)**	8	/	
** L2 (colonic)**	2	/	
** L3 (ileocolonic)**	8	/	
** L4 (upper disease)**	1	/	
**Disease behavior^a^ [CD], *n* ^a^**			**–**
** B1 (nonstricturing, nonpenetrating)**	8	/	
** B2 (stricturing)**	6	/	
** B3 (penetrating)**	4	/	
**Concomitant therapy at enrolment**			**–**
** Mesalamine, *n*°**	**–**	20	
** Thiopurines, *n*°**	1	0	
** Corticosteroids, *n*°**	**–**	5	
**Previous anti-TNF treatment, *n*°**	16	15	
** More than 1 anti-TNF, *n*°**	11	6	
**Mayo score at baseline, mean ± SD**	**–**	9 ± 2	**–**
**Mayo endoscopic subscore at baseline, mean ± SD**	**–**	2 ± 0.5	**–**
**HBI at baseline (mean ± SD)**	7 ± 4	**–**	**–**
**SES-CD at baseline (mean ± SD)**	9 ± 4	**–**	**–**

Abbreviations: CD, Crohn’s disease; HBI, Harvey–Bradshaw Index; IBD, inflammatory bowel disease; *n*, number of subjects; SD, standard deviation; SES-CD, simple endoscopic score for Crohn’s disease; TNF, tumor necrosis factor; UC, ulcerative colitis.

a According to the Montreal classification.

### Bulk RNA-sequencing data analysis

Data was obtained from our previously published work^2^ and analyzed following the same workflow. Briefly, raw counts were normalized using DESeq2 (R v4.1.1), and differentially expressed genes were identified based on a false discovery rate (FDR) below 5% and a log2 fold change greater than 1.

### scRNA-sequencing data analysis

The scRNA-sequencing data were obtained from Colonna and colleagues (GSE157477)^10^ and analyzed using Scanpy v1.9.7. Quality control steps included filtering cells and genes based on gene abundance < 500 and > 3000, expression levels in at least 1% of all single cells, mitochondrial and ribosomal gene content >10% and <10%, respectively. To isolate CD3^+^CD4^+^T-cells, we selected cells with zero expression values of CD8A and CD8B and excluded a γδ T-cell cluster identified by the expression of TRDC, TRGC1, and TRGC2. A total of 24 771 CD4^+^T-lymphocytes were retained. Batch effects arising from multiple donors were corrected using Harmony tool (v0.0.10). Clustering was performed using the sc.tl.leiden function with a resolution parameter set to 1.0. The resulting clusters were manually annotated based on known marker gene expression and the results from Differential Gene Expression analysis (sc.tl.rank_genes_groups). Gene set Enrichment Analysis (GSEA) was performed using gp.preprank function from GSEApy v1.1.9. Gene set scores were calculated using the sc.tl.score genes function.

### Flow cytometry

Peripheral blood mononuclear cells (PBMC) were isolated by density gradient centrifugation by Ficoll density gradient (Amersham Pharmacia Biotech, Uppsala, Sweden). Lamina propria mononuclear cells (LPMCs) and intraepithelial lymphocytes (IELs) were isolated as previously described.^11^ Mesenteric lymph nodes (LNs) were smashed into 70-μm nylon strainers (BD Biosciences) and erythrocytes lysed with red blood cell (RBC) lysis buffer (BD Biosciences). For characterization of T helper (Th)-cell subsets from PBMCs, LNs, LPMCs, and IELs, T cells were stained with a combination of fluorochrome-conjugated antibodies as reported previously^2^ and Th17 cells gated as CD4^+^IL-7R^+^CD25^low^CCR6^+^CXCR3^-^ cells; they were further subdivided according to CCR5 expression into CCR5^+^“pTh17”- and CCR5^-^“cTh17”-cells. Th17-cell subsets were analyzed for the expression of CD103, CD69, CCR9, and α4/β7-integrin by staining with fluorochrome-conjugated monoclonal antibodies. Intracellular cytokines in gated CCR6^+^CD4^+^T-cells were detected after stimulation with PMA and Ionomycin for 5 hours in the presence of Brefeldin A as reported previously.^2^ Analysis was performed with a FACSCanto II cytometer (Becton Dickinson, Franklin Lakes, NJ, United States) and analyzed using FlowJo software (BD Biosciences).

### ROC curves

Receiver operating characteristic (ROC) curves and area under the curve (AUC) values were computed using pROC package on R (v. 4.3.3). The optimal cutoff point for each model was determined using Youden’s index (J = sensitivity + specificity − 1), which identifies the threshold that maximizes the sum of sensitivity and specificity. In order to integrates pTh17 levels measured in both LPL and IEL compartments, we applied a logistic regression and generated a composite score reflecting the combined contribution of the 2 compartments. Since in this case the threshold incorporates information from both LPL and IEL, it is inherently two-dimensional and it has been represented as a decision boundary line in the two-dimensional marker space.

### Statistics

Statistical significance was evaluated using paired or unpaired *t*-test for comparison of 2 groups (Mann—Whitney’s or Welch’s correction were applied to analyze variables not normally distributed) or by one-way ANOVA for comparison of more than 2 groups (Dunn’s multiple comparisons to analyze variables that were not normally distributed). Significance was defined at *P* < .05. Statistics were performed with Prism software (version 7; GraphPad Software, Inc., La Jolla, CA, United States).

## 3. Results

### Intestinal pTh17-cells contain tissue-resident memory cells (T_RM_) and become activated in the intestinal intraepithelial lymphocyte compartment

**Figure 1.**
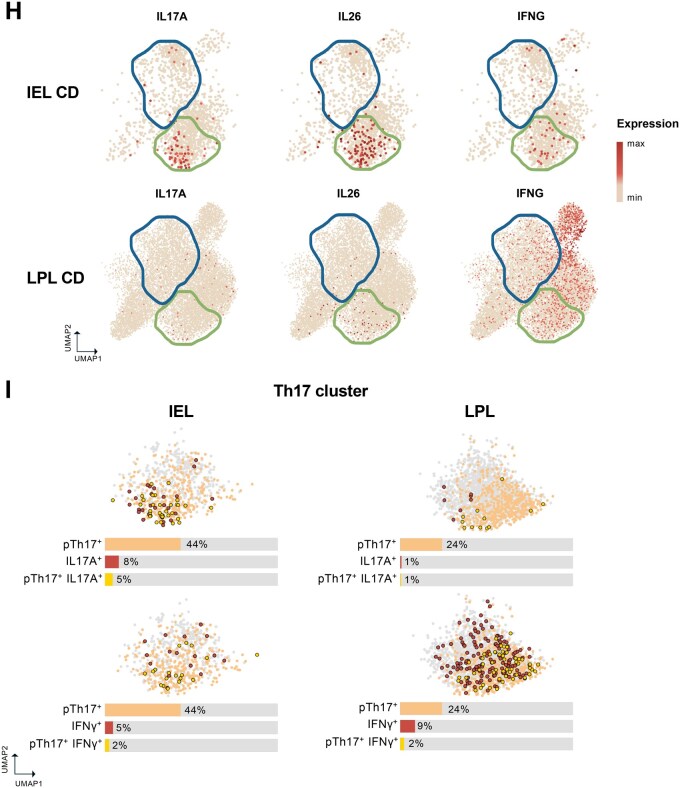

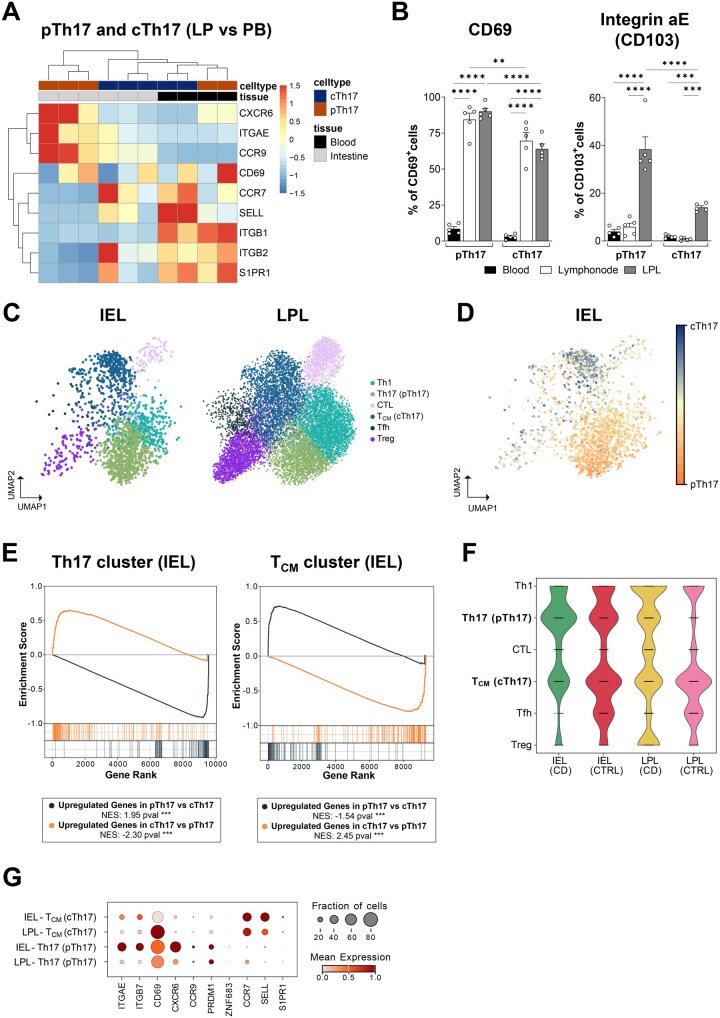
Intestinal pTh17-cells display features of TRM and are enriched among IELs in CD patients. (A) Heatmap of genes regulating tissue residency and recirculation in cTh17- and pTh17-cells sequenced in bulk from the blood and the intestine al LP. (B**)** Ex-vivo expression of CD69 and CD103 in gated T-cell subsets from peripheral blood (black bars), intestinal LNs (white bars) or the intestinal LP (gray bars) from CD patients (*n* = 6). Statistical significance was calculated using one-way ANOVA and reported as *P < 0.05, **P < 0.01, ***P < 0.001, ****P < 0.0001. (C). UMAPs of single RNA-sequenced T-cells show CD4^+^T-cell clusters among IEL (left) or LP lymphocytes (LPL). Six clusters were annotated according to signature genes (Figure S1C, see online supplementary material for a color version of this figure). The color code is illustrated in the panel. (D) The UMAP shows the scaled gene set scores for the pTh17 and cTh17 signature derived from bulk RNAseq, among IELs. Orange cells express pTh17-associated gens and blue cells cTh17-associated genes. (E) GSEA using the DEGs of LP pTh17-cells (orange line) or LP cTh17-cells (blue line) for the intraepithelial Th17 cluster (left) or in the intraepithelial T_CM_ cluster (right). (F) Relative frequencies of the 6 CD4^+^T-cell clusters among LPL and IEL in CD or control patients (CTRL). (G) Dot plots show expression levels of genes implicated in T-cell residency and recirculation in the Th17 and TCM clusters from LPL and IEL from CD patients. (H) UMAPs showing cytokine expression in the IEL and LPL compartments of CD patients. The T_CM_ and Th17 clusters are highlighted by blue and green lines, respectively. (I) Cytokine-expressing cells in the Th17 clusters of IEL or LPL were highlighted and analyzed for the expression of pTh17-associated genes. Cytokine-expressing cells that scored positive for the pTh17-cell signature are indicated in yellow and those that scored negative in red. Cells that did not express cytokines but scored positive for the pTh17-cell signature are indicated in orange and those that scored negative in gray. Percentages are referred to total number of cells in the Th17 cluster.

We previously performed bulk RNA sequencing of Th17 subsets from the blood and the LP of CD patients.^2^ We analyzed here the expression of genes that regulate tissue residency and recirculation in CCR5^+^pTh17-cells and conventional CCR5^-^Th17-cells (“cTh17”) from the LP and the blood (Figure 1A and Figure S1A, see online supplementary material for a color version of this figure). LP pTh17-cells expressed significantly higher levels of genes promoting tissue residency, including ITGAE (integrin-αE, CD103), as compared to circulating pTh17-cells and to LP cTh17-cells. In contrast they expressed significantly lower levels of genes involved in recirculation, namely S1PR1 (Sphingosine-1 Receptor 1), SELL (L-Selectin), ITGB1, ITGB2, and CCR7.^2^ Notably, LP cTh17-cells clustered closer to circulating Th17 subsets then to LP pTh17-cells (Figure 1A). CD103 and CD69 expression are characteristic of tissue-resident memory T-cells (T_RM_). Consistently, CD69 surface expression was hardly detectable in the blood but was very high on intestinal LNs and in LP (Figure 1B). Notably, virtually all LP pTh17-cells expressed the tissue retention marker CD69 and approximately 40% co-expressed CD103 (Figure 1B and Figure S1B, see online supplementary material for a color version of this figure). In accordance with gene expression analysis, significantly smaller fractions of LP cTh17-cells expressed CD69 and CD103.

Since CD103 binds to E-cadherin on epithelial cells, we investigated if pTh17-cells were enriched in the intraepithelial compartment. We interrogated a single-cell RNA-sequencing dataset of total intestinal T-cells from the LP and the intraepithelial compartment of CD patients.^10^ We first generated UMAPs of CD4^+^T-cells from the LP and the epithelium and identified 6 clusters that were annotated as Th1, Th17, cytotoxic (CTL), central memory (T_CM_),^12^ follicular helper (Tfh) and regulatory T-cells (Tregs) according to signature genes (Figure 1C and Figure S1C and D, see online supplementary material for a color version of this figure). To understand how pTh17-cells and cTH17-cells were distributed among the identified clusters, we performed a Gene Score analysis with the genes that were differentially expressed in bulk-sequenced LP Th17 subsets (Figure S1A, see online supplementary material for a color version of this figure). Interestingly, pTh17 and cTh17-cells were separated into different clusters (Figure 1D). Thus, intraepithelial cells expressing pTh17-associated genes were enriched in the Th17-cluster, whereas cells that expressed cTh17-associated genes were enriched in the T_CM_ cluster. GSEA confirmed a highly significant enrichment of pTh17 signature genes in the Th17 cluster and of cTh17 signature genes in the T_CM_ cluster (Figure 1E). Also in the LP cells expressing pTh17- and cTh17-associated genes were separated and significantly enriched in, respectively, the Th17- and T_CM_ clusters (Figure S1E and F, see online supplementary material for a color version of this figure). Notably, the pTh17-enriched Th17 clusters were more abundant among IELs and in CD (Figure 1F). Conversely, the cTh17-enriched T_CM_ clusters were more abundant in the control patients. We then analyzed the expression of genes regulating tissue residency and recirculation in the T_CM_ and Th17 clusters of CD patients from the LP and IELs (Figure 1G). Intraepithelial pTh17-enriched Th17 cluster expressed the highest levels of the tissue residency-associated genes CXCR6, ITGAE, and ITGB7 (coding for integrin αEβ7). Conversely, genes promoting recirculation like CCR7, CD62L and S1PR1 were largely restricted to the cTh17-enriched T_CM_ clusters. The analysis of T_RM_-associated transcription factors^8^ unveiled that PRDM1 (Blimp1) was expressed in Th17- but not in T_CM_ clusters, whereas ZNF683 (Hobit) was hardly detectable. Intriguingly, some cells in the intraepithelial Th17 cluster expressed IL-17A and IL-26 (Figure 1H), consistent with the notion that they were activated in vivo.^10^ Cells in the Th17 cluster of the LP expressed lower levels of type 17 cytokines, but they expressed the Th17-associated chemokine CCL20 (Figure S1F, see online supplementary material for a color version of this figure). IL-2 and IFN-γ expression were most prominent among Th1 and cytotoxic clusters of the LP, respectively, but some IFN-γ was also detected in the Th17 clusters (Figure 1H). Conversely, cytokine-expressing cells were rare in the T_CM_ clusters. To investigate if the activated cells in the Th17 clusters corresponded to pTh17-cells, we analyzed the expression of pTh17-associated genes of cytokine-expressing cells (Figure 1I). In the intraepithelial Th17 cluster, 8% of cells expressed IL-17A, and the majority of these IL-17A^+^cells scored positive for the pTh17-associated gene signature. In the LP only 1% of cells in the Th17 cluster expressed IL-17A, but these IL-17A^+^cells were virtually all positive for the pTh17 gene score. Notably, IL-17A^+^cells in the LP and among IELs scored in contrast negative for the cTh17 gene score (Figure S1G, see online supplementary material for a color version of this figure). Finally, 2% of cells in the intraepithelial and LP Th17 clusters with a positive pTh17 score expressed IFNG, consistent with the fact that pTh17-cells can produce both IL-17 and IFN-γ.^2^

In summary, these results suggest that pTh17-cells are gut-resident cells that home to the intestinal epithelium and become activated to produce effector cytokines.

### pTh17-cells recirculate poorly, are not efficiently targeted by vedolizumab, and predict unresponsiveness to therapy

**Figure 2.**
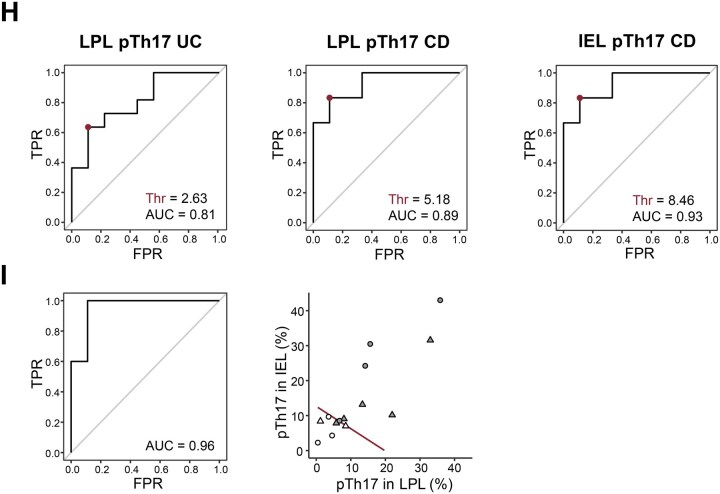

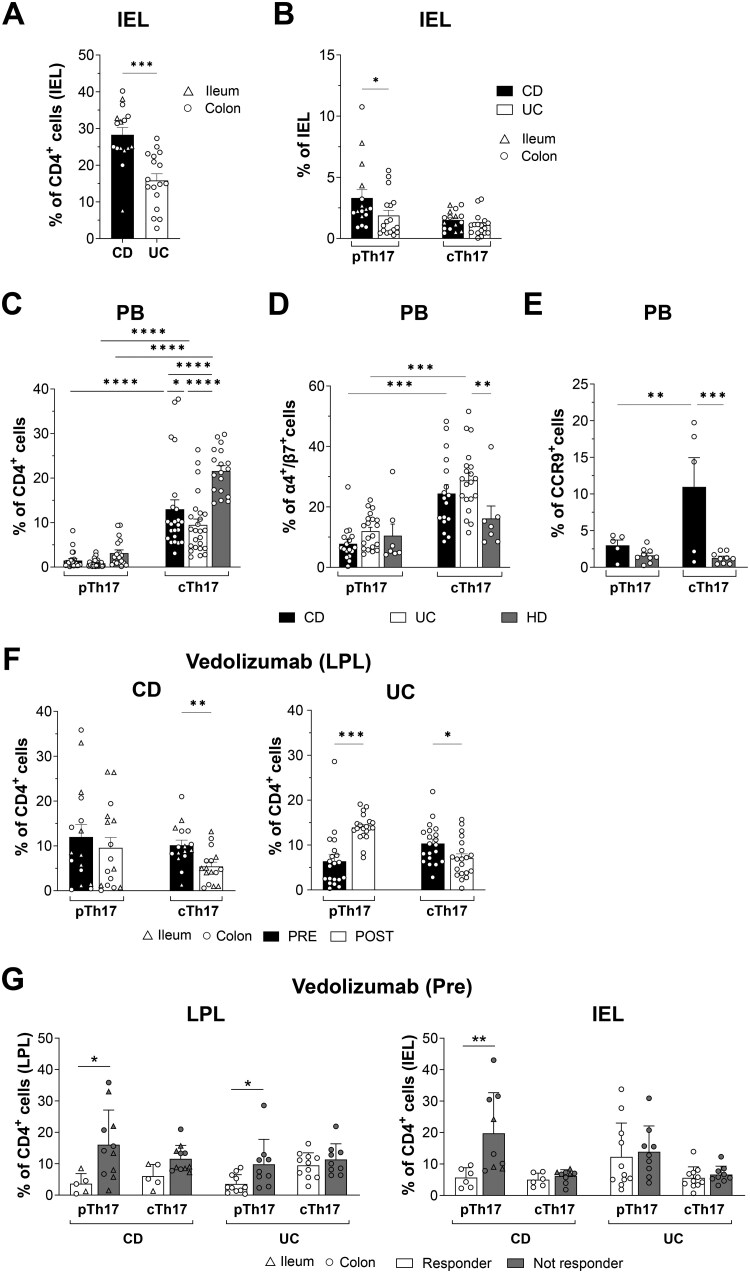
pTh17-cells recirculate poorly and are associated with unresponsiveness to vedolizumab. Percentages of (A) CD4+T-cells (left) and (B) of Th17-cell subsets (right) among intestinal intraepithelial lymphocytes (IEL) of CD (*n* = 14) and UC (*n* = 15) patients. (C) Frequencies of Th17-cell subsets in peripheral blood of HDs (*n* = 19), CD (*n* = 24) and UC patients (*n* = 25). (D) Percentage of α4β7-integrin expressing cells among the Th17-cell subsets in peripheral blood of HD (*n* = 7), UC (*n* = 20) and CD patients (*n* = 16). (E) Ex-vivo expression of CCR9 in Th17 subsets from peripheral blood of HD and CD patients (*n* = 6). (F) Frequencies of Th17-cell subsets among CD4^+^T-cells in the intestinal LP at baseline (t = 0) and after 14 weeks of vedolizumab treatment (t = 14) of CD (*n* = 16) and UC patients (*n* = 20). (G) Frequencies of Th17 subsets among CD4^+^T-cells in the intestinal LP (left) or in the intraepithelial compartment (right) at baseline of vedolizumab treatment in responding and nonresponding CD (*n* = 5 and 11) and UC (*n* = 11 and 9) patients. Statistical significances were calculated using one-way ANOVA and reported as *P < 0.05, **P < 0.01, ***P < 0.001, ****P < 0.0001. (A) In the left graph an unpaired *t*-test was used. (H) ROC curves for pTh17 frequencies in LPL in UC (left), LPL in CD (center) and IEL in CD (right). The threshold derived from the Youden’s index is indicated in red. (I) ROC curves resulting from the logistic regression model integrating both LPL and IEL pTh17 frequencies of CD patients. On the right, the decision boundary and the corresponding thresholds (THR) for responder and not responder are indicated in red.

To precisely assess the frequencies of intraepithelial pTh17-cells in IBDs we then analyzed Th17-cell subsets from the LP and among IELs in intestinal biopsies of IBD patients (Table 1) by flow cytometry. CD patients contained a significant higher fraction of CD4^+^T-cells among IELs as compared to UC patients (Figure 2A). This was true for CD patients with either ileal or colonic inflammation (Figure S2A, see online supplementary material for a color version of this figure). Moreover, pTH17- but not cTh17-cells were significantly increased among IELs in CD (Figure 2B and Figure S2B, see online supplementary material for a color version of this figure).

Next, to investigate if intestinal pTh17-cells were tissue-resident or could recirculate, we analyzed circulating Th17-cell subsets for the expression of α4β7-integrin (Figure S2C, see online supplementary material for a color version of this figure), which is critical for gut homing.^13^ cTh17-cells were abundant in the blood, but they were significantly reduced in CD and UC patients^2^ as compared to healthy donors (HD, Figure 2C), possibly because they were recruited to the gut. Circulating pTh17-cells were in contrast rare, in particular in IBD patients. Moreover, only a small fraction pTh17-cells expressed of α4β7-integrin (Figure 2D and Figure S2D, see online supplementary material for a color version of this figure), indicating that circulating pTh17-cells with gut-homing capacities are extremely rare (Figure S2E, see online supplementary material for a color version of this figure).

A higher percentage of cTh17 cells expressed α4β7-integrin in HDs and a significantly higher fraction was α4β7-integrin^+^in IBDs (Figure 2D), suggesting a relevant intestinal trafficking capacity that increased in IBDs. We also analyzed surface expression of CCR9 (Figure S2C, see online supplementary material for a color version of this figure), which promotes homing to the small intestine. The percentages of CCR9^+^cells was indeed significantly increased on cTh17-cells (Figure 2E) in a chort of ileal CD patients.^2^ Conversely, pTh17-cells contained only low fractions of CCR9^+^cells, consistent with a low gut-homing potential.

To further investigate the intestinal trafficking capacities of Th17 subsets, we monitored their dynamics in IBD patients upon treatment with vedolizumab (Table 1), which blocks α4β7-integrin and lymphocyte homing to the gut.^9^^,^^14^^,^^15^ Importantly, the frequencies of cTh17-cells were significantly reduced by vedolizumab in the LP in CD and UC patients (Figure 2F). Conversely, α4β7^+^cTh17-cells increased in the blood of CD patients post-vedolizumab treatment (Figure S2E, see online supplementary material for a color version of this figure), suggesting that gut-homing cTh17-cells were retained in the circulation. Circulating α4β7^+^pTh17-cells remained in contrast hardly detectable post-vedolizumab treatment (Figure S2E, see online supplementary material for a color version of this figure).

These results suggested that circulating pTh17-cells were not efficiently targeted by vedolizumab. Moreover, in a limited number of IBD patients we observed that unresponsive individuals contained significantly higher frequencies of CCR6^+^T-cells that could produce IL-17, IFN-γ and TNF upon polyclonal stimulation (Figure S2F, see online supplementary material for a color version of this figure), ie, proinflammatory cytokines that are produced, although not exclusively, by pTh17-cells.^2^ We therefore analyzed if preexisting intestinal pTh17-cells might be associated with unresponsiveness to vedolizumab therapy. Indeed, the frequencies of pTh17-cells pre-therapy were significantly higher in the LP and among CD4^+^IELs in CD patients that failed to respond to vedolizumab therapy (Figure 2G). Surprisingly, also unresponsive UC patients had significantly increased frequencies of pre-existing pTh17-cells in the LP (Figure 2G), but the frequencies were as expected lower as compared to unresponsive CD patients.^2^ Moreover, intraepithelial pTh17-cells were not increased in unresponsive UC patients. No significant associations were detected for preexisting intestinal cTh17-cells. Notably, α4β7^+^cTh17-cells were significantly increased in the blood and significantly decreased in the LP and among IELs post-vedolizumab treatment in unresponsive patients (Figure S2E, see online supplementary material for a color version of this figure), suggesting that intestinal T-cell homing was successfully blocked. Finally, ROC curve analysis was done to identify predictive thresholds for patient classification into responders and non-responders. In UC, a pTh17 percentage below 2.6% among CD4^+^LPL corresponded to responders (AUC = 0.81, Figure 2H, Table S1). The thresholds and AUC values were higher for CD patients, ie, 5.2% among CD4^+^LPL (AUC = 0.89) and 8.5% among CD4^+^IEL (AUC = 0.93). To integrate pTh17 frequencies in both compartments, we used a logistic regression model and generated a composite score that was superior to identify non-responders (AUC = 0.96, Figure 2I). It was represented as a decision boundary line in a two-dimensional marker space. The logistic regression coefficients (LPL = −0.74 and IEL = −1.16) indicate that IEL contributed more to determine the decision boundary. These thresholds may provide a guideline to classify patients as responders or non-responders based on their pTh17 frequencies in the intestine in independent cohorts (Table S1).

In summary, these results indicate that intestinal pTh17 cells are T_RM_ that recirculate poorly, are consequently not efficiently targeted by vedolizumab and their baseline frequencies in the gut predict unresponsiveness to therapy. Conversely, cTh17-cells, ie, their presumed precursors, possessed an increased gut-homing potential in the blood of IBD patients and decreased in the intestine upon vedolizumab treatment, suggesting that they are highly relevant cellular targets.

## 4. Discussion

We previously reported that pTh17-cells are a proinflammatory Th17 effector population that were abundant in the intestinal LP, associated with intestinal inflammation in CD and activated by AIEC, consistent with a key pathogenic role.^2^ Here we showed that pTh17-cells express genes and surface receptors of tissue-resident cells (T_RM_) and were enriched in the intraepithelial compartment in CD. Conversely, pTh17-cells with gut-homing potential were hardly detectable in the blood, and were absent from intestinal LNs,^2^ consistent with the concept that intestinal pTh17-cells are T_RM_ that are largely unable to recirculate.^8^ A pathogenic role for T_RM_ in IBDs is consistent with previous reports,^8^^,^^16^ but these reports did not address the role of pTh17-cells. In CD a role for T_RM_ is supported by the slow kinetics of vedolizumab therapy and its lower efficiency in CD patients,^9^ because Vedolizumab blocks gut-homing of recirculating T-cells,^14^^,^^15^^,^^17^ but spare preformed intestinal T_RM_. Consistently, patients with relatively high frequencies of preexisting intestinal pTh17-cells failed to respond to Vedolizumab.

The finding that CCR5^+^pTh17-cells were enriched among T_RM_ and IELs in CD is consistent with a role for CCR5 in intestinal inflammation^18^^,^^19^ and the notion that T-cell homing to CD-associated epithelial granulomas is promoted by the CCR5 ligand RANTES.^20^ AIEC, which are associated with CD and specifically activated pTh17-cells, induced RANTES production by dendritic cells and are involved in the formation of granulomas.^2^^,^^21^ Moreover, AIEC could infect epithelial cells, possibly explaining why pTh17-cells were activated to express IFN-γ and IL-17 in the intraepithelial compartment.

Since circulating pTh17-cells with gut-homing potential are extremely rare, pTh17_RM_-cells are probably derived from other precursors. These precursors cells are likely to be cTh17-cells, which are T_CM_^5^^,^^12^ that are quite abundant in intestinal LNs^2^ and in peripheral blood and possessed increased gut-homing potentials in IBDs. Consistently, previous reports suggested that Vedolizumab targets circulating CD4^+^T-cells, including Th17-cells.^9^^,^^14^^,^^15^ Principle component analysis of the transcriptomes of cTh17 and pTh17-cells suggested indeed that they are distantly related.^2^ Moreover, TCR stimulation of cTh17-cells in the presence of IL-23 induced the phenotype and cytokine profile of pTh17-cells in vitro.^5^^,^^22^ Consistently, blocking IL-23 with Risankizumab resulted in a selective reduction of intestinal pTh17-cells in vivo. Indeed, IL-23 is a key colitogenic cytokine that induces Th17-cell maturation^23^ and acts directly on T-cells to induce colitis in mice.^24^

Collectively, our data are thus consistent with a two-step model where intestinal pTh17_RM_ cells are generated from recirculating cTh17_CM_-cells with gut-homing capacities. These precursors are presumably primed in intestinal LNs, migrate then via the blood to the gut and further differentiate to pTh17 cells upon activation by DC that present antigens derived from colitogenic bacteria like AIEC and secrete IL-23.^2^ This model is inconsistent with a previous report which concluded T-cell trafficking is not affected by Vedolizumab treatment.^17^^,^^25^ Nevertheless, it has relevant implications for the pathogenesis of CD. Indeed, we provided proof of principle here that monitoring intestinal pTh17-cells might help to predict the unresponsiveness to vedolizumab. Consistently, a recent report showed that non-responders to Vedolizumab therapy contained proliferating α4/β1-expressing CD4^+^memory T-cells with a Th1/Th17 phenotype in the blood.^26^ Finally, pTh17_RM_-cells might be exploited as immunological targets for the future development of therapeutic strategies for Vedolizumab-resistant CD.

## Supplementary Material

jjag055_Supplementary_Data

## Data Availability

The scRNA-sequencing data in Figure 1 are publicly available (GSE157477).^10^ The raw data of Figure 2 are available upon reasonable request to the corresponding authors.
