# Supplementary material for: Pro-inflammatory intestinal Th17-cells are tissue-resident, accumulate in the epithelia in Crohn’s disease, and predict unresponsiveness to vedolizumab
Source: J Crohns Colitis. 2026 Jun 9;20(6):jjag055. doi: 10.1093/ecco-jcc/jjag055 (PMC13250732; doi:10.1093/ecco-jcc/jjag055)
Supplement: jjag055_Supplementary_Data [file jjag055_supplementary_data.pdf]

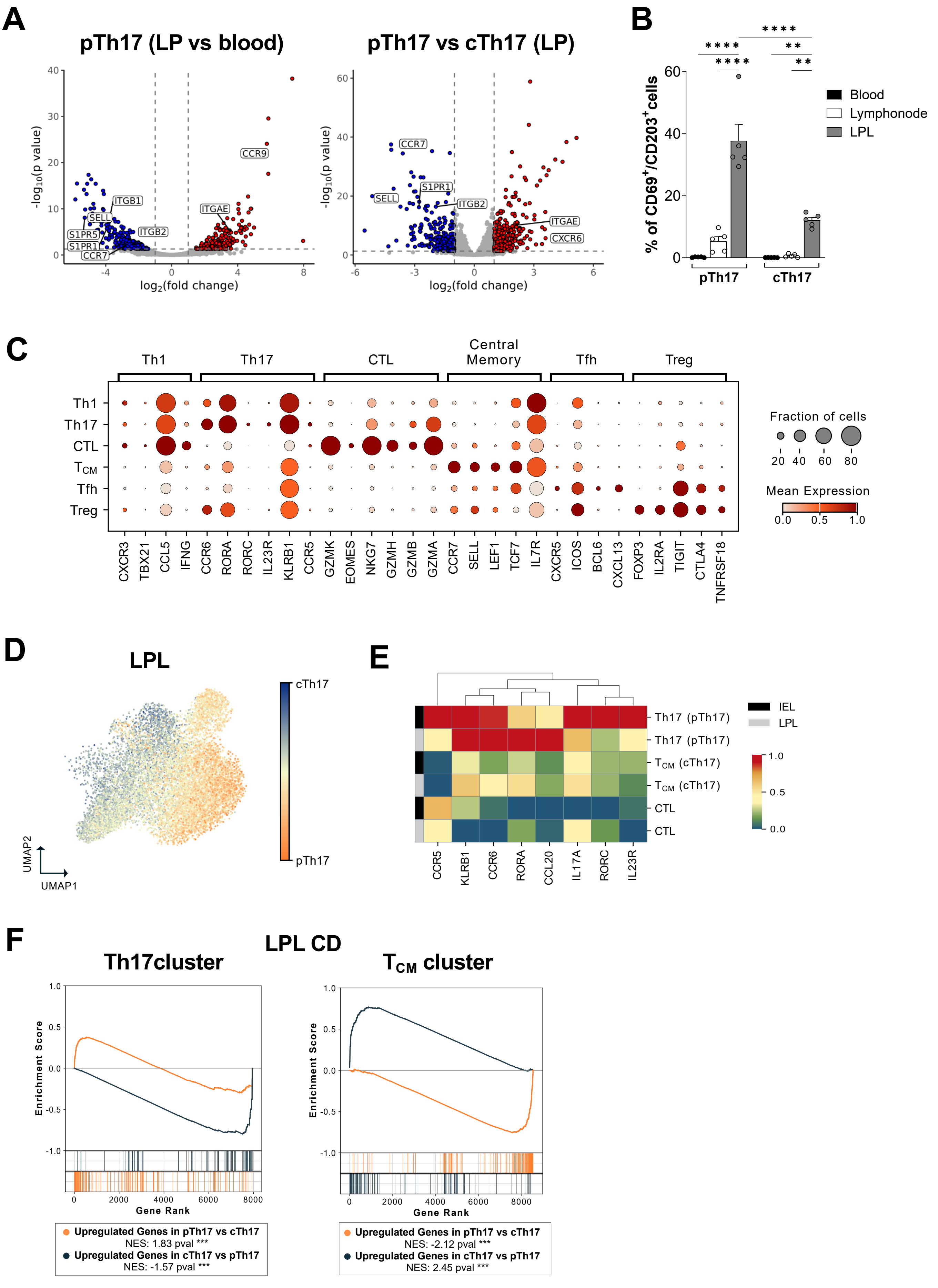
A
pTh17 (LP vs blood)
pTh17 vs cTh17 (LP)
-log10(p value)
log2(fold change)
CCR9
ITGB1
SELL
S1PR5
S1PR1
CCR7
ITGB2
ITGAE
SELL
S1PR1
ITGB2
CXCR6
B
% of CD69+/CD203+cells
Blood
Lymphonode
LPL
pTh17
cTh17
C
Th1
Th17
CTL
Central Memory
Tfh
Treg
TCM
Fraction of cells
20 40 60 80
Mean Expression
0.0 0.5 1.0
CXCR3
TBX21
CCL5
IFNG
CCR6
RORA
RORC
IL23R
KLRB1
CCR5
GZMK
EOMES
NKG7
GZMH
GZMB
GZMA
CCR7
SELL
LEF1
TCF7
IL7R
CXCR5
ICOS
BCL6
CXCL13
FOXP3
IL2RA
TIGIT
CTLA4
TNFRSF18
D
LPL
cTh17
pTh17
UMAP1
UMAP2
E
IEL
LPL
Th17 (pTh17)
TCM (cTh17)
CTL
CCR5
KLRB1
CCR6
RORA
CCL20
IL17A
RORC
IL23R
F
LPL CD
Th17cluster
TCM cluster
Enrichment Score
Gene Rank
Upregulated Genes in pTh17 vs cTh17
NES: 1.83 pval ***
Upregulated Genes in cTh17 vs pTh17
NES: -1.57 pval ***
Upregulated Genes in pTh17 vs cTh17
NES: -2.12 pval ***
Upregulated Genes in cTh17 vs pTh17
NES: 2.45 pval ***

sFigure 1

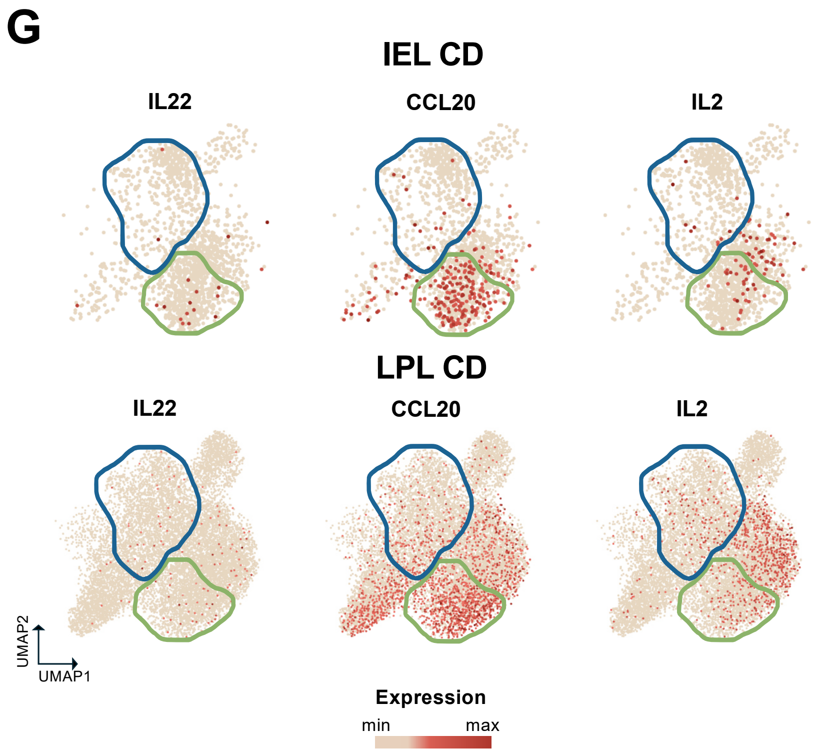
G
IEL CD
IL22
CCL20
IL2
LPL CD
IL22
CCL20
IL2
UMAP2
UMAP1
Expression
min
max

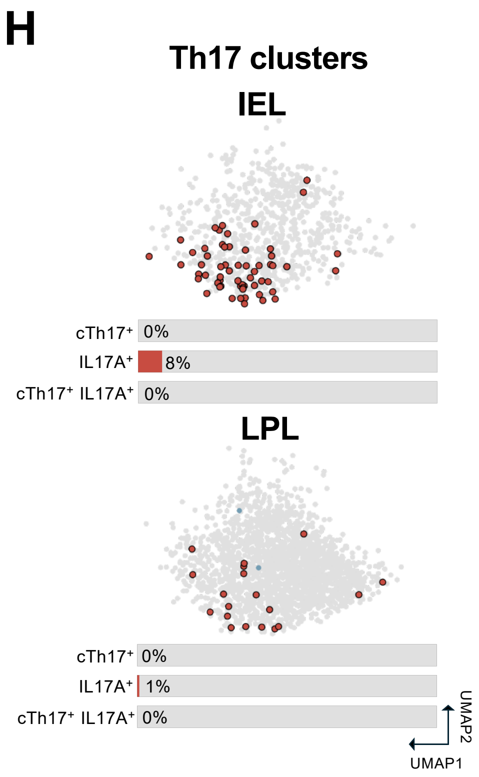
H
Th17 clusters
IEL
cTh17⁺ 0%
IL17A⁺ 8%
cTh17⁺ IL17A⁺ 0%
LPL
cTh17⁺ 0%
IL17A⁺ 1%
cTh17⁺ IL17A⁺ 0%
UMAP2
UMAP1

sFigure 1

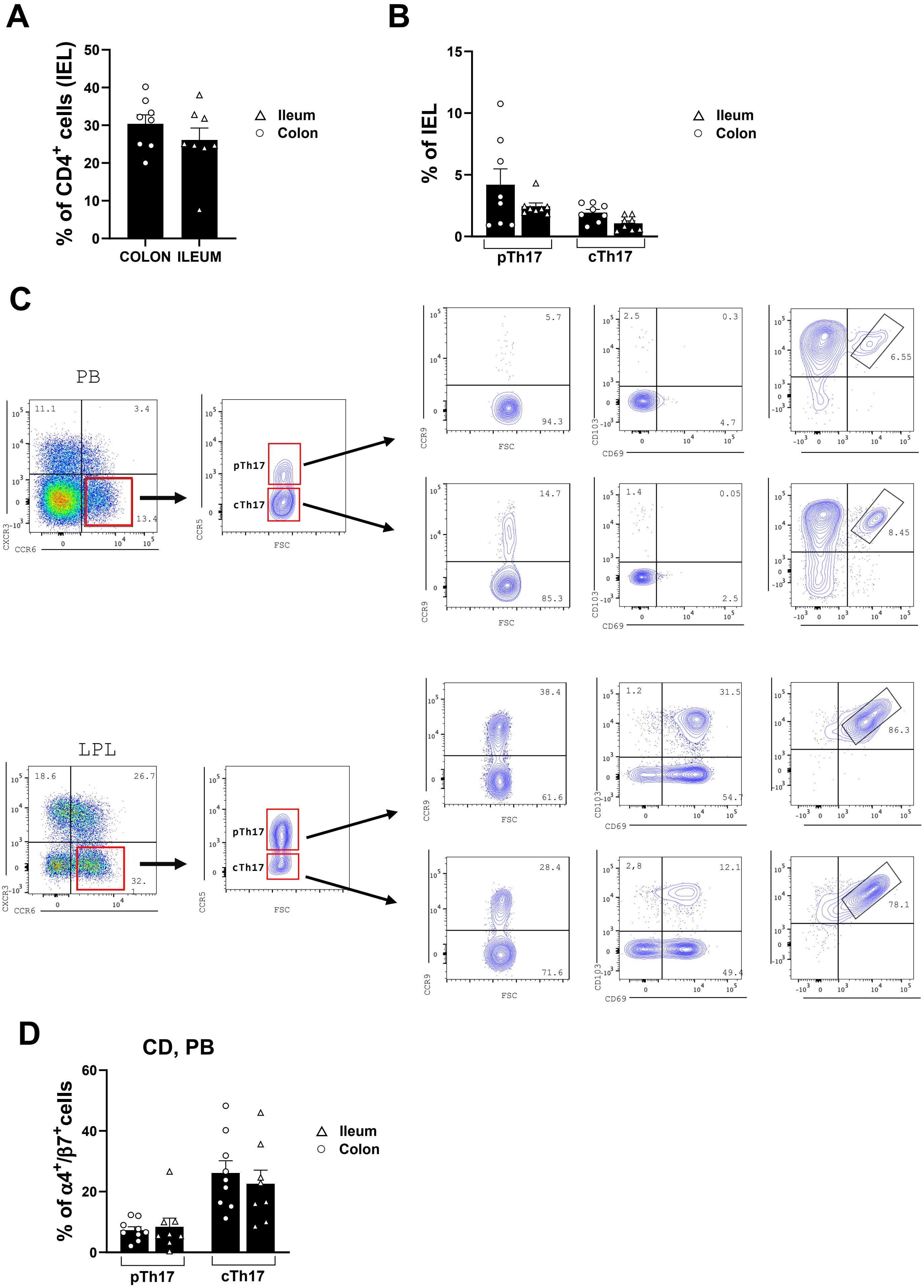
A
% of CD4+ cells (IEL)
COLON ILEUM
Ileum
Colon
B
% of IEL
pTh17
cTh17
Ileum
Colon
C
PB
LPL
pTh17
cTh17
CXCR3
CCR6
CCR5
FSC
CCR9
CD103
CD69
D
CD, PB
% of α4+/β7+cells
pTh17
cTh17
Ileum
Colon

sFigure 2

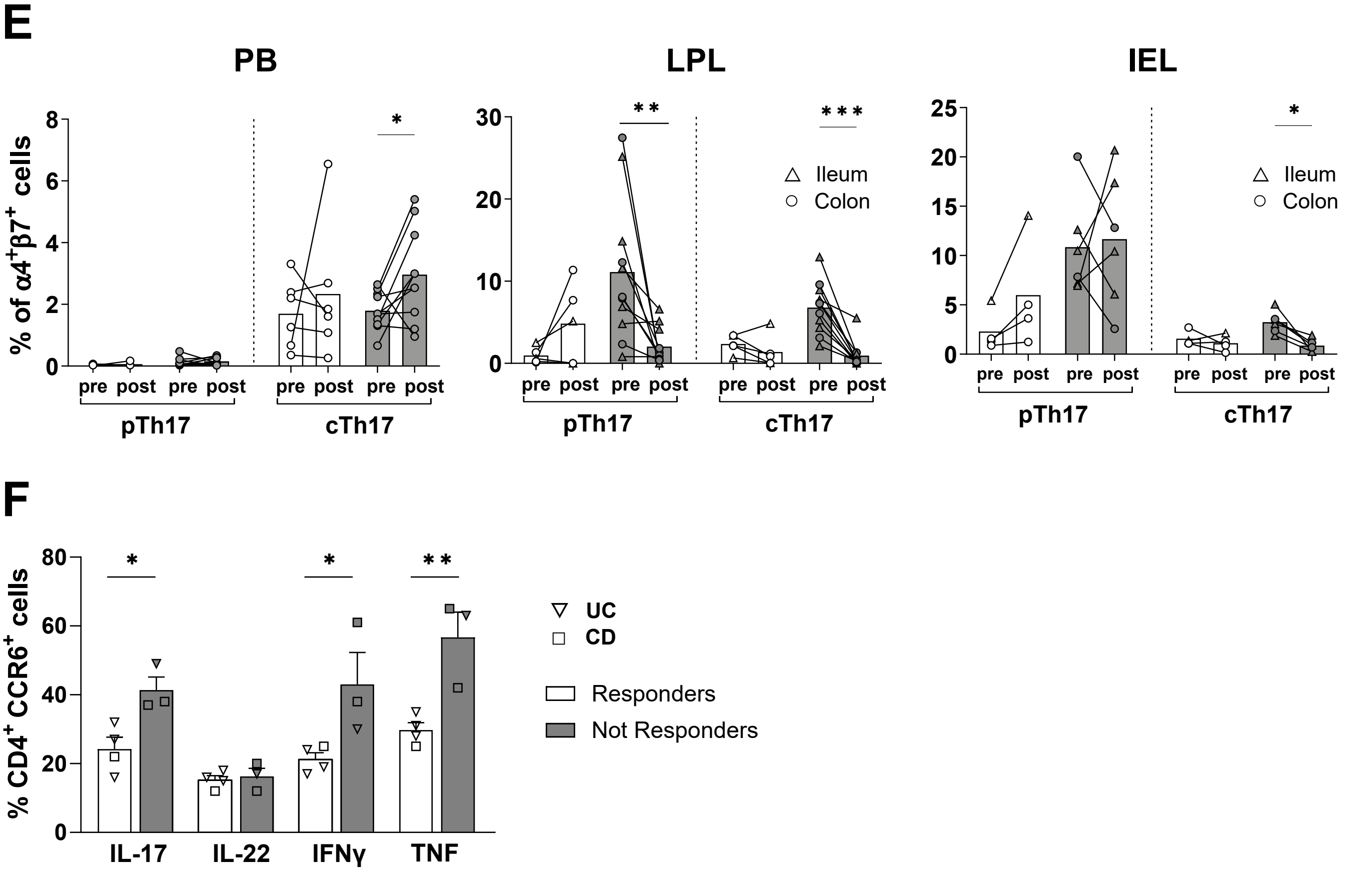
E
PB
LPL
IEL
% of α4+β7+ cells
pre post pre post pre post pre post
pTh17
cTh17
*
**
***
Ileum
Colon
F
% CD4+ CCR6+ cells
IL-17
IL-22
IFNγ
TNF
UC
CD
Responders
Not Responders

sFigure 2

sTable 1

	LPL pTh17 UC	LPL pTh17 CD	IEL pTh17 CD	LR
**AUC**	0.81 (0.61 – 1)	0.89 (0.72 – 1)	0.93 (0.79 – 1)	0.96 (0.85 – 1)
**Estimated Threshold**	2.63	5.18	8.46	/
**Sensitivity**	0.64 (0.31 – 0.89)	0.80 (0.28 – 0.99)	0.83 (0.36 – 1)	1 (0.48 – 1)
**Specificity**	0.89 (0.52 – 1)	0.91 (0.59 – 1)	0.89 (0.52 – 1)	0.89 (0.52 – 1)
**PPV**	0.88 (0.47 – 1)	0.8 (0.28 – 0.99)	0.83 (0.36 – 1)	0.83 (0.36 – 1)
**NPV**	0.67 (0.35 – 0.9)	0.91 (0.59 – 1)	0.89 (0.52 – 1)	1 (0.63 – 1)
